# Outdoor physical activity, mental health, life satisfaction, happiness and life stress among Canadian adolescents

**DOI:** 10.24095/hpcdp.45.7/8.02

**Published:** 2025-08

**Authors:** Taylor Bradbury, Justin J. Lang, Stephanie A. Prince, Gary S. Goldfield, Louise de Lannoy, Mark S. Tremblay, Jean-Philippe Chaput

**Affiliations:** 1 Department of Health Sciences, Carleton University, Ottawa, Ontario, Canada; 2 Centre for Surveillance and Applied Research, Public Health Agency of Canada, Ottawa, Ontario, Canada; 3 School of Epidemiology and Public Health, Faculty of Medicine, University of Ottawa, Ottawa, Ontario, Canada; 4 Healthy Active Living and Obesity Research Group, Children’s Hospital of Eastern Ontario Research Institute, Ottawa, Ontario, Canada; 5 Alliance for Research in Exercise, Nutrition and Activity (ARENA), Allied Health and Human Performance, University of South Australia, Adelaide, Australia; 6 Department of Pediatrics, Faculty of Medicine, University of Ottawa, Ottawa, Ontario, Canada; 7 Outdoor Play Canada, Ottawa, Ontario, Canada

**Keywords:** physical activity, outdoor time, youth, lifestyle, psychological health, public health, adolescence

## Abstract

**Introduction:**

The objective of this article is to examine the association between outdoor physical activity (OPA) and mental health, life satisfaction, happiness and life stress among Canadian adolescents aged 12 to 17 years.

**Methods:**

This cross-sectional and nationally representative study used self-reported data from the 2019 Canadian Health Survey on Children and Youth (n=10413). The survey categorized OPA into six groups (from 0 to ≥14 hours/week). Logistic regression analyses examined the associations between OPA levels and outcomes, with adjustments for relevant covariates.

**Results:**

In adjusted models, OPA was not significantly associated with anxiety or depressive symptoms. Compared to adolescents with no OPA, those who engaged in ≥14 hours/week had higher odds of positive mental health (odds ratio [OR] = 1.64; 95% confidence interval [CI]: 1.13–2.38), high life satisfaction (OR = 1.75; 95% CI: 1.24–2.46) and high happiness (OR = 2.36; 95% CI: 1.59–3.50), independent of covariates including indoor physical activity. A positive dose–response relationship was observed between higher levels of OPA and life satisfaction and happiness.

**Conclusion:**

Independent of indoor physical activity and other covariates, OPA was associated with positive mental health, high life satisfaction and high happiness, with levels of OPA of ≥14 hours/week (highest category) showing the strongest associations. Further studies are needed to elucidate the mechanisms linking OPA with higher life satisfaction and happiness.

**Highlights**

- Adolescence is an age when mental health may decline. Many adolescents in Canada are also insufficiently physically active.
- Outdoor physical activity (OPA) may provide added health benefits compared to indoor physical activity, but adolescents are spending less time outdoors.
- Independent of indoor physical activity, OPA was associated with positive mental health, high life satisfaction and high happiness among adolescents.
- 14 or more hours per week of OPA had the strongest associations with positive mental health, high life satisfaction and high happiness.
- There was a clear dose–response relationship between higher levels of OPA and life satisfaction and happiness.

## Introduction

Mental health refers to an individual’s emotional, psychological and social well-being.^1^ According to the Mental Health Commission of Canada, approximately 1.2million children and adolescents are affected by mental illness, and 70% of adults with mental illness experienced symptoms before they were 18 years old.^2^ As such, understanding the factors that contribute to adolescents’ mental health is essential. The most common mental health issues among Canadian adolescents are anxiety (e.g. social anxiety disorder, specific phobias, performance anxiety) and depression.^3^

Positive mental health is the capacity to feel, think and act in ways that enhance the ability to enjoy life and deal with challenges.^4^ Adolescents with high positive mental health are able to function well across different settings, experience happiness, cope well with life stress and enjoy a positive quality of life.^4^ Adolescence is also a critical stage for developing behavioural, social and emotional habits—such as regular physical activity—that support long-term mental well-being.^5^

Mental health is complex and multifaceted, and an array of factors contribute to both positive and negative outcomes. For example, positive mental health (e.g. flourishing, resiliency) is associated with a physically active lifestyle and good sleep habits, whereas poor mental health is associated with excessive sedentary behaviour and screen time, physical inactivity, unhealthy diet and poor sleep patterns.^6^ Avoiding mental illness and promoting good mental health therefore requires various strategies. Exploring positive mental health indicators can help improve adolescent mental health.

A behaviour that may contribute to adolescents’ positive mental health is participation in outdoor physical activity (OPA). Physical activity is defined as any bodily movement produced by skeletal muscles that requires energy expenditure.^7^ OPA is any form of physical activity that occurs in any open-air, wild, natural or human-made outdoor space.^8^ Physical activity is essential for adolescents’ healthy development and has been associated with physical, social and mental health benefits.^9^ Yet, according to the 2024 ParticipACTION Report Card on Physical Activity for Children and Youth, only 39% of children and youth in Canada are meeting the recommendation of 60 minutes per day of moderate-to-vigorous intensity physical activity.^10^ The Canadian 24-Hour Movement Guidelines for Children and Youth^11^ recommend that indoor time be replaced with outdoor time, but do not specify the amount of outdoor time because research in this area is scarce. Interacting with nature is positively associated with the mental health of children and youth (although the associations were not found to be consistently significant).^12^ Interactions with nature, which can occur during outdoor activities, may also be associated with lower stress levels.^12^,^13^ Compared with previous generations, adolescents spend less time in nature nowadays.^14^ In addition, adolescence is a period when time spent outdoors and being physically active typically decrease and mental health also declines.^15^

Although the association between physical activity and mental health of children and adolescents in Canada has been widely investigated, there has been little research on OPA and mental health.^16^,^17^ OPA has been less studied than indoor activity due to the challenges of controlling environmental variables like weather, terrain, air quality and social settings, which can complicate data collection and analysis. Also, few studies have explored the association between adolescent stress levels and interactions with the outdoors; additional research is needed to confirm any benefits.^12^,^18^ Therefore, it is important to better understand the relationship between OPA and various mental health indicators, including anxiety, depressive symptoms, life satisfaction, happiness and life stress in adolescents.

Implementing treatments, interventions and prevention strategies for mental health issues among adolescents requires taking into account the specific problems they face, which are distinct from those experienced by adults. A better understanding of the connection between OPA and adolescents’ mental health is important to help understand and develop important targets for intervention strategies and inform public health policies. Further, because various mental health problems begin in adolescence, identifying early life interventions can help prevent problems later in life.^2^,^19^ Thus, this study addresses important knowledge gaps to better inform the development of future interventions.

The objective of this study was to investigate associations between OPA and self-perceived mental health, symptoms of anxiety and depression, life satisfaction, happiness and life stress among Canadian adolescents in a large and nationally representative sample. We hypothesized that greater levels of OPA would be associated with better mental health indicators after adjusting for indoor physical activity and other relevant covariates.

## Methods

***Study design and participants***

This cross-sectional and nationally representative study used data from the 2019 Canadian Health Survey on Children and Youth (CHSCY). The CHSCY, which was conducted by Statistics Canada, collected data from 11 February to 2 August 2019. Detailed information about the survey methodology is available elsewhere.^20^ In brief, the target population for the 2019 CHSCY was children and youth aged 1 to 17 years residing in the 10 provinces and the three territories of Canada. The Canada Child Benefit was used to create the survey frame. Excluded from the survey’s coverage were children and youth living on First Nation reserves and other Indigenous settlements and in foster homes and institutions. Approximately 98% of the children and youth aged 1 to 17 years in the provinces and 96% of those in the territories were included in the survey frame. The present study focuses on adolescents aged 12 to 17 years because the OPA question was not used in the survey for children younger than 12years old.

The adolescent participants answered survey questions directly through an online electronic questionnaire, or through telephone interview for follow-up on nonresponses. The 2019 CHSCY dataset had a total response rate of 52.1%, yielding a sample of 11077 participants aged 12 to 17 years. For the present analysis, respondents lacking information on OPA (n=36), outcome measures (n=167) or covariate information (n=461) were excluded, resulting in a final sample size of 10413 participants.

Statistics Canada secured the necessary approvals to conduct the CHSCY. Pursuant to Article 2.2 of the Tri-Council Policy Statement on Ethical Conduct for Research Involving Humans ( https://ethics.gc.ca/eng/policy-politique_tcps2-eptc2_2022.html), Statistics Canada’s CHSCY data are considered publicly available information through a mechanism set out by legislation or regulation that is protected by law and therefore their use for research purposes does not require review by a research ethics board, as long as there is no linkage to other datasets. Informed consent from participants was obtained before they participated in the study.

***Independent variable: outdoor physical activity (OPA)***

Participants were asked about OPA in the past 7 days. Participants responded with either “yes” or “no” to the first question: “In the past 7 days, did you participate in any outdoor physical activities in your free time, such as biking, skating, gardening, playing ball or sledding?” Those who answered “no” were coded as having no OPA. Those who responded “yes” were then asked, “In the past 7 days, how much time did you spend participating in these outdoor physical activities in your free time?” The five response options (<1hour; 1hour to < 3 hours; 3 hours to < 7 hours; 7 hours to < 14 hours; and ≥14 hours) to this question and the “no OPA” response to the first question were used for analysis.

***Dependent variables: perceived mental health, symptoms of anxiety and depression, life satisfaction, happiness and life stress***

Based on availability in the CHSCY, we included indicators of mental wellness and illness. Self-perceived mental health was assessed with the question “In general, how is your mental health?” The response options were “excellent,” “very good,” “good,” “fair” and “poor.” Responses of “excellent” and “very good” were coded as having high (positive) mental health, in accordance with the Positive Mental Health Surveillance Indicator Framework (PMHSIF).^21^,^22^ Self-perceived mental health is a valid and widely used indicator in population health surveys associated with multi-item measures of mental health, self-rated health and health-related problems.^23^

Anxiety and depressive symptoms were assessed using validated questions from the Washington Group/UNICEF Child Functioning Module.^24^-^27^ Anxiety symptoms were assessed using the question “How often do you seem very anxious, nervous or worried?” Depressive symptoms were assessed using the question, “How often do you seem very sad or depressed?” Response options included “daily,” “weekly,” “monthly,” “a few times a year” and “never.” Responses of “a few times a year” and “never” were coded as having low anxiety or depressive symptoms.^21^

Life satisfaction was measured with the following item: “Using a scale of 0 to 10, where 0 means ‘very dissatisfied’ and 10 means ‘very satisfied,’ how do you feel about your life as a whole right now?” For our study, we dichotomized life satisfaction as “high life satisfaction” (score ≥9), based on the PMHSIF.^21^ Perceived life satisfaction is routinely used as an indicator of social well-being, and many studies have supported its validity.^28^,^29^ Perceived mental health significantly influences life satisfaction.^30^

Self-perceived happiness was assessed by asking participants whether they would usually describe themselves as “happy and interested in life,” “somewhat happy,” “somewhat unhappy,” “unhappy with little interest in life” or “so unhappy that life is not worthwhile.” The response of “happy and interested in life” was coded as high self-perceived happiness. Single-item happiness measures have shown good validity in adolescents, and happiness is associated with positive health and healthier development during adolescence.^31^,^32^

Finally, self-perceived life stress was assessed by asking participants how they would describe the amount of stress in their life on most days. Response options included “not at all stressful,” “not very stressful,” “a bit stressful,” “quite a bit stressful” and “extremely stressful.” Responses of “not at all stressful,” “not very stressful” and “a bit stressful” were coded as having low life stress, in line with Skinner et al.’s contextual analysis.^33^ Perceived life stress is another important factor affecting population health, and single-item assessments have demonstrated comparability to more extensive questionnaires in gauging perceived general life stress.^34^

***Covariates***

Age (in years), sex (male or female), highest parental education level (from less than high school to graduate university degree), ethnocultural background (14 options), average sleep duration (hours per night), total recreational screen time (from no recreational screen time to ≥21 hours/week), data collection season (winter, spring, summer), urbanicity (urban, rural) and indoor physical activity (from no indoor physical activity to ≥14 hours in the past week) were used as covariates in the analyses based on their availability in the dataset and their known associations in the literature with the outcome measures.

***Statistical analysis***

Comparisons of positive mental health, low anxiety and depressive symptoms, high life satisfaction, high levels of happiness (or “high happiness”) and low life stress between sex (male versus female) and age groups (12–14 and 15–17 years) were undertaken through chi-square tests. Logistic regression analyses were conducted to examine the associations between levels of OPA and the outcome measures, with adjustment for covariates. Odds ratios (OR) and 95% confidence intervals (CI) are reported. Statistics Canada–derived sample weights were applied to address the survey’s sampling design and potential nonresponse bias to ensure that our findings remain representative of the broader adolescent population in Canada. To account for survey design effects, bootstrap weights were utilized to estimate 95% CI.

All statistical analyses were conducted using statistical package SAS Enterprise Guide 7.1 (SAS Institute Inc., Cary, NC, US).

## Results

Of the adolescents in Canada, 36% reported no OPA and only 3% reported 14 or more hours per week (Table 1). The most noticeable difference was in the prevalence of high happiness among adolescents with no OPA (54.4%) and those with 14 or more hours per week of OPA (81.5%). There were several significant within-group differences in OPA for positive mental health, high life satisfaction, high happiness and low life stress. There were also many between-group significant differences by sex and age for most outcome measures.

**Table 1:** Prevalence of perceived mental health, anxiety and depressive symptoms, life satisfaction, happiness and life stress based on levels of OPA among adolescents aged 12–17 years, by sex and age group, Canada, 2019 (n = 10 413)

Variable	% (95% CI)					
	0 h/wk (n = 3783)	< 1 h/wk (n = 727)	1 to < 3 h/wk (n = 2700)	3 to < 7 h/wk (n = 2116)	7 to < 14 h/wk (n = 787)	≥ 14 h/wk (n = 300)
Positive mental health						
Total sample	60.1 (58.0–62.2)	64.8 (60.0–69.6)	69.0 (66.6–71.3)	71.9 (69.4–74.3)	74.6 (70.4–78.7)	78.0 (72.2–83.8)^*^
Male	69.3 (66.3–72.4)^a^	71.8 (65.3–78.2)^a^	75.6 (72.6–78.5)^a^	77.1 (74.1–80.1)^a^	78.8 (74.2–83.4)^a^	83.2 (76.4–90.0)^a^
Female	53.0 (50.2–55.8)	58.7 (51.8–65.6)	62.1 (58.4–65.8)	64.3 (60.3–68.4)	65.4 (57.6–73.2)	68.2 (56.7–79.6)
12–14 years	69.9 (67.1–72.8)^b^	73.5 (68.1–78.9)^b^	75.2 (72.5–78.0)^b^	77.5 (74.5–80.5)^b^	82.1 (78.0–86.2)^b^	83.5 (77.4–89.6)^b^
15–17 years	53.4 (50.6–56.2)	54.1 (46.2–61.9)	60.4 (56.4–64.5)	64.3 (60.2–68.4)	59.8 (51.8–67.9)	70.4 (59.5–81.3)
Low anxiety symptoms						
Total sample	67.7 (65.7–69.7)	67.2 (62.6–71.8)	70.4 (68.2–72.7)	72.1 (69.5–74.7)	69.0 (64.8–73.2)	68.2 (61.5–74.9)
Male	77.2 (74.5–79.9)^a^	72.6 (66.3–79.0)^a^	76.5 (73.4–79.7)^a^	77.6 (74.5–80.8)^a^	72.5 (67.5–77.4)^a^	70.6 (62.3–79.0)
Female	60.4 (57.7–63.2)	62.5 (55.7–69.3)	64.1 (60.6–67.6)	64.2 (60.0–68.4)	61.4 (53.5–69.4)	63.7 (52.4–75.0)
12–14 years	71.3 (68.3–74.3)^b^	66.4 (60.5–72.4)	73.1 (70.3–75.9)^b^	72.5 (69.1–75.9)	70.6 (65.6–75.6)	67.8 (59.2–76.4)
15–17 years	65.2 (62.6–67.9)	68.2 (61.3–75.0)	66.9 (63.0–70.7)	71.6 (67.8–75.3)	65.8 (58.3–73.4)	68.8 (58.4–79.3)
Low depressive symptoms						
Total sample	83.0 (81.4–84.5)	82.2 (78.2–86.1)	85.5 (83.7–87.3)	85.7 (83.8–87.5)	86.3 (83.0–89.5)	85.1 (80.2–90.0)
Male	88.1 (86.1–90.1)^a^	88.4 (84.0–92.8)^a^	89.1 (86.8–91.5)^a^	90.2 (88.1–92.4)^a^	87.5 (83.6–91.3)	87.2 (81.4–92.9)
Female	79.0 (76.7–81.3)	76.7 (70.6–82.9)	81.8 (79.1–84.5)	79.1 (75.7–82.6)	83.7 (77.7–89.7)	81.3 (72.1–90.5)
12–14 years	87.3 (85.4–89.3)^b^	83.7 (79.0–88.3)	87.0 (84.7–89.3)	86.4 (84.1–88.8)	85.7 (81.5–89.9)	88.6 (83.1–94.2)
15–17 years	80.0 (77.7–82.2)	80.4 (73.9–86.8)	83.5 (80.7–86.3)	84.7 (81.6–87.8)	87.4 (82.4–92.3)	80.3 (71.6–88.9)
High life satisfaction						
Total sample	37.5 (35.3–39.6)	42.9 (37.9–47.8)	47.9 (45.4–50.5)	51.1 (48.2–54.1)	57.0 (52.4–61.5)	62.3 (55.4–69.3)^*^
Male	40.3 (37.0–43.6)^a^	44.6 (37.6–51.7)	49.6 (46.2–53.0)	54.3 (50.5–58.1)^a^	60.8 (55.5–66.2)^a^	65.0 (56.7–73.3)
Female	35.3 (32.4–38.1)	41.3 (34.4–48.2)	46.2 (42.4–49.9)	46.5 (41.9–51.2)	48.6 (40.6–56.5)	57.4 (45.2–69.6)
12–14 years	46.0 (42.7–49.4)^b^	53.2 (46.6–59.8)^b^	55.2 (52.0–58.4)^b^	60.0 (56.1–63.9)^b^	64.4 (59.0–69.7)^b^	68.4 (60.5–76.3)
15–17 years	31.6 (28.8–34.4)	30.2 (23.1–37.2)	37.9 (34.0–41.9)	39.2 (35.0–43.3)	42.5 (34.5–50.5)	54.0 (41.5–66.5)
High happiness						
Total sample	54.4 (52.3–56.5)	62.8 (57.9–67.6)	68.3 (65.9–70.7)	73.7 (71.0–76.3)	76.4 (72.6–80.1)	81.5 (75.9–87.1)^*^
Male	57.0 (53.6–60.3)^a^	67.5 (60.6–74.4)	70.2 (67.0–73.5)	75.8 (72.5–79.2)	77.8 (73.5–82.1)	85.0 (79.6–90.4)
Female	52.4 (49.5–55.2)	58.7 (51.8–65.6)	66.2 (62.7–69.8)	70.5 (66.3–74.7)	73.3 (65.9–80.7)	75.0 (63.1–86.9)
12–14 years	61.6 (58.5–64.7)^b^	73.8 (68.6–79.1)^b^	71.4 (68.4–74.4)^b^	77.1 (73.8–80.4)^b^	80.0 (75.7–84.3)^b^	86.9 (81.7–92.1)^b^
15–17 years	49.4 (46.6–52.3)	49.2 (41.3–57.0)	64.1 (60.1–68.0)	69.0 (64.9–73.1)	69.3 (62.0–76.5)	74.0 (63.3–84.7)
Low life stress						
Total sample	74.7 (72.9–76.5)	76.6 (72.3–81.0)	82.7 (80.7–84.7)	84.9 (82.8–87.0)	83.1 (79.4–86.8)	83.1 (77.0–89.2)^*^
Male	83.3 (80.9–85.7)^a^	84.7 (79.7–89.6)^a^	88.6 (86.3–90.9)^a^	89.4 (87.0–91.7)^a^	87.6 (83.9–91.3)^a^	86.6 (79.8–93.4)
Female	68.1 (65.5–70.7)	69.7 (63.2–76.1)	76.5 (73.2–79.8)	78.5 (74.8–82.2)	73.2 (65.4–81.0)	76.6 (64.7–88.5)
12–14 years	83.6 (81.3–85.8)^b^	83.8 (79.2–88.4)^b^	87.1 (84.8–89.3)^b^	90.1 (87.7–92.5)^b^	88.6 (84.8–92.5)^b^	93.9 (90.0–97.8)^b^
15–17 years	68.6 (66.0–71.3)	67.8 (60.1–75.5)	76.6 (73.1–80.1)	77.9 (74.4–81.5)	72.2 (64.7–79.7)	68.3 (56.4–80.3)

**Source: **Canadian Health Survey on Children and Youth, 2019. 

**Abbreviations:** CI, confidence interval; h, hour; OPA, outdoor physical activity; wk, week. 

**Notes:** A chi-square test was used to compare proportions between sex and age groups. Positive mental health includes responses of “excellent” and “very good.” Low anxiety or depressive symptoms include responses of “a few times a year” and “never.” High life satisfaction includes scores ≥ 9. High happiness includes responses of “happy and interested in life.” Low life stress includes responses of “not at all stressful,” “not very stressful” and “a bit stressful.”

^a^ Males are significantly different from females (*p* < 0.05). 

^b^ Youth aged 12–14 years are significantly different from youth aged 15–17 years (*p* < 0.05). 

* There is a significant difference within the sample (*p* < 0.05). 

In the fully adjusted models, OPA was not significantly associated with anxiety or depressive symptoms, and largely showed null associations with life stress (Table 2). However, compared to adolescents with no OPA (the reference group), those who engaged in 14 or more hours per week of OPA had higher odds of positive mental health (adjusted odds ratio [aOR] = 1.64; 95% CI: 1.13–2.38), high life satisfaction (aOR = 1.75; 95% CI: 1.24–2.46) and high happiness (aOR = 2.36; 95% CI: 1.59–3.50), independent of indoor physical activity time and other covariates. There were also clear dose–response associations for high life satisfaction and high happiness.

**Table 2:** Associations between levels of OPA and mental health, anxiety and depressive symptoms, life satisfaction, happiness and life stress among all adolescents (12–17 years), Canada, 2019 (n = 10 413)

OPA level, h/wk	Unadjusted OR (95% CI)	Adjusted OR (95% CI)
Positive mental health		
0	1.00 [Reference]	1.00 [Reference]
< 1	1.22 (0.97–1.55)	1.01 (0.79–1.30)
1 to < 3	1.48 (1.28–1.70)	1.09 (0.94–1.27)
3 to < 7	1.70 (1.46–1.97)	1.18 (1.00–1.39)
7 to < 14	1.95 (1.53–2.48)	1.19 (0.92–1.53)
≥ 14	2.34 (1.65–3.32)	1.64 (1.13–2.38)
Low anxiety symptoms		
0	1.00 [Reference]	1.00 [Reference]
< 1	0.98 (0.78–1.22)	0.97 (0.77–1.23)
1 to < 3	1.14 (0.98–1.32)	1.09 (0.94–1.27)
3 to < 7	1.24 (1.06–1.45)	1.13 (0.95–1.34)
7 to < 14	1.06 (0.85–1.31)	0.92 (0.73–1.16)
≥ 14	1.02 (0.75–1.41)	0.87 (0.62–1.24)
Low depressive symptoms		
0	1.00 [Reference]	1.00 [Reference]
< 1	0.95 (0.70–1.26)	0.92 (0.68–1.23)
1 to < 3	1.21 (1.01–1.45)	1.08 (0.89–1.31)
3 to < 7	1.23 (1.02–1.48)	1.04 (0.85–1.28)
7 to < 14	1.29 (0.96–1.73)	1.03 (0.75–1.42)
≥ 14	1.17 (0.78–1.76)	0.97 (0.63–1.48)
High life satisfaction		
0	1.00 [Reference]	1.00 [Reference]
< 1	1.25 (1.00–1.57)	0.99 (0.78–1.25)
1 to < 3	1.54 (1.34–1.76)	1.10 (0.95–1.28)
3 to < 7	1.74 (1.51–2.02)	1.22 (1.04–1.44)
7 to < 14	2.21 (1.80–2.71)	1.41 (1.14–1.75)
≥ 14	2.75 (2.01–3.77)	1.75 (1.24–2.46)
High happiness		
0	1.00 [Reference]	1.00 [Reference]
< 1	1.41 (1.13–1.77)	1.20 (0.95–1.52)
1 to < 3	1.81 (1.57–2.08)	1.36 (1.17–1.58)
3 to < 7	2.35 (2.00–2.74)	1.73 (1.46–2.05)
7 to < 14	2.72 (2.16–3.43)	1.82 (1.43–2.32)
≥ 14	3.70 (2.52–5.43)	2.36 (1.59–3.50)
Low life stress		
0	1.00 [Reference]	1.00 [Reference]
< 1	1.11 (0.85–1.45)	0.89 (0.68–1.17)
1 to < 3	1.62 (1.36–1.92)	1.15 (0.96–1.39)
3 to < 7	1.91 (1.58–2.30)	1.28 (1.04–1.58)
7 to < 14	1.66 (1.26–2.20)	0.92 (0.69–1.22)
≥ 14	1.67 (1.05–2.61)	0.99 (0.64–1.54)

**Source: **Canadian Health Survey on Children and Youth, 2019. 

**Abbreviations:** CI, confidence interval; h, hours; OPA, outdoor physical activity; OR, odds ratio; wk, week.

**Notes:** Logistic regression models were used to examine the associations between OPA and the outcome measures. Models were adjusted for age, sex, highest parental education, ethnocultural background, average sleep duration (hours/night), total recreational screen time (hours/week), season, urbanicity and indoor physical activity (categories from no indoor physical activity to ≥ 14 hours/week). 

Positive mental health includes responses of “excellent” and “very good” (vs. “good,” “fair” and “poor”). Low anxiety symptoms and low depressive symptoms include responses of “a few times a year” and “never” (vs. “daily,” “weekly” and “monthly”). High life satisfaction includes scores ≥ 9 on a scale of 0–10 (vs. scores < 9 for low life satisfaction). High happiness includes responses of “happy and interested in life.” Low life stress includes responses of “not at all stressful,” “not very stressful” and “a bit stressful” (vs. “quite a bit stressful” and “extremely stressful”). 

Subgroup analyses stratified by sex demonstrated similar and stronger overall associations for males versus females and younger versus older adolescents (12–14 years versus 15–17 years) (Tables 3–6).

**Table 3:** Associations between levels of OPA and mental health, anxiety and depressive symptoms, life satisfaction, happiness and life stress among male adolescents (12–17 years), Canada, 2019 (n = 5109)

OPA level, h/wk	Unadjusted OR (95% CI)	Adjusted OR (95% CI)
Positive mental health		
0	1.00 [Reference]	1.00 [Reference]
< 1	1.12 (0.79–1.60)	0.99 (0.69–1.43)
1 to < 3	1.37 (1.10–1.71)	1.06 (0.84–1.34)
3 to < 7	1.49 (1.18–1.87)	1.17 (0.92–1.49)
7 to < 14	1.64 (1.20–2.24)	1.24 (0.89–1.73)
≥ 14	2.17 (1.29–3.65)	1.90 (1.12–3.25)
Low anxiety symptoms		
0	1.00 [Reference]	1.00 [Reference]
< 1	0.78 (0.55–1.13)	0.85 (0.58–1.24)
1 to < 3	0.97 (0.76–1.22)	1.02 (0.80–1.30)
3 to < 7	1.03 (0.81–1.31)	1.07 (0.83–1.38)
7 to < 14	0.78 (0.58–1.04)	0.86 (0.63–1.17)
≥ 14	0.71 (0.46–1.09)	0.72 (0.46–1.13)
Low depressive symptoms		
0	1.00 [Reference]	1.00 [Reference]
< 1	1.03 (0.64–1.67)	1.14 (0.69–1.86)
1 to < 3	1.10 (0.81–1.50)	1.11 (0.82–1.52)
3 to < 7	1.25 (0.91–1.72)	1.27 (0.91–1.77)
7 to < 14	0.94 (0.62–1.42)	1.04 (0.68–1.61)
≥ 14	0.92 (0.53–1.61)	1.00 (0.55–1.82)
High life satisfaction		
0	1.00 [Reference]	1.00 [Reference]
< 1	1.19 (0.86–1.64)	1.01 (0.72–1.43)
1 to < 3	1.45 (1.19–1.76)	1.10 (0.89–1.37)
3 to < 7	1.75 (1.43–2.15)	1.37 (1.09–1.72)
7 to < 14	2.29 (1.76–2.98)	1.69 (1.27–2.25)
≥ 14	2.72 (1.84–4.03)	1.86 (1.20–2.88)
High happiness		
0	1.00 [Reference]	1.00 [Reference]
< 1	1.56 (1.11–2.20)	1.43 (1.00–2.04)
1 to < 3	1.77 (1.44–2.19)	1.45 (1.16–1.81)
3 to < 7	2.36 (1.89–2.94)	1.98 (1.56–2.50)
7 to < 14	2.64 (1.99–3.51)	2.07 (1.52–2.83)
≥ 14	4.29 (2.73–6.74)	3.04 (1.87–4.96)
Low life stress		
0	1.00 [Reference]	1.00 [Reference]
< 1	1.10 (0.71–1.71)	0.98 (0.62–1.56)
1 to < 3	1.55 (1.16–2.07)	1.21 (0.89–1.63)
3 to < 7	1.68 (1.24–2.29)	1.35 (0.97–1.89)
7 to < 14	1.41 (0.96–2.09)	1.05 (0.70–1.59)
≥ 14	1.28 (0.68–2.39)	1.00 (0.53–1.88)

**Source:** Canadian Health Survey on Children and Youth, 2019.

**Abbreviations: **CI, confidence interval; h, hour; OPA, outdoor physical activity; OR: odds ratio; wk, week.

**Notes:** Logistic regression models were used to examine the associations between OPA and the outcome measures. Models were adjusted for age, highest parental education, ethnocultural background, average sleep duration (hours/night), total recreational screen time (hours/week), season, urbanicity and indoor physical activity (categories from no indoor physical activity to ≥ 14 hours/week).

Positive mental health includes responses of “excellent” and “very good” (vs. “good,” “fair” and “poor”). Low anxiety symptoms and low depressive symptoms include responses of “a few times a year” and “never” (vs. “daily,” “weekly” and “monthly”). High life satisfaction includes scores ≥ 9 on a scale of 0–10 (vs. scores < 9 for low life satisfaction). High happiness includes responses of “happy and interested in life.” Low life stress includes responses of “not at all stressful,” “not very stressful” and “a bit stressful” (vs. “quite a bit stressful” and “extremely stressful”).

**Table 4:** Association between levels of OPA and mental health, anxiety and depressive symptoms, life satisfaction, happiness and life stress among female adolescents (12–17 years), Canada, 2019 (n = 5304)

OPA level, h/wk	Unadjusted OR (95% CI)	Adjusted OR (95% CI)
Positive mental health		
0	1.00 [Reference]	1.00 [Reference]
< 1	1.26 (0.92–1.73)	1.05 (0.75–1.45)
1 to < 3	1.45 (1.20–1.76)	1.11 (0.89–1.37)
3 to < 7	1.60 (1.30–1.97)	1.22 (0.96–1.54)
7 to < 14	1.69 (1.16–2.45)	1.10 (0.75–1.61)
≥ 14	1.90 (1.10–3.28)	1.41 (0.76–2.61)
Low anxiety symptoms		
0	1.00 [Reference]	1.00 [Reference]
< 1	1.09 (0.79–1.48)	1.05 (0.76–1.45)
1 to < 3	1.17 (0.97–1.42)	1.17 (0.95–1.43)
3 to < 7	1.18 (0.95–1.46)	1.18 (0.93–1.50)
7 to < 14	1.03 (0.72–1.48)	1.07 (0.74–1.54)
≥ 14	1.15 (0.69–1.91)	1.17 (0.68–2.01)
Low depressive symptoms		
0	1.00 [Reference]	1.00 [Reference]
< 1	0.86 (0.59–1.26)	0.80 (0.55–1.16)
1 to < 3	1.19 (0.94–1.49)	1.09 (0.85–1.38)
3 to < 7	1.00 (0.78–1.29)	0.91 (0.69–1.19)
7 to < 14	1.36 (0.85–2.17)	1.19 (0.72–1.97)
≥ 14	1.15 (0.61–2.17)	1.07 (0.55–2.09)
High life satisfaction		
0	1.00 [Reference]	1.00 [Reference]
< 1	1.29 (0.94–1.77)	0.98 (0.71–1.35)
1 to < 3	1.58 (1.30–1.91)	1.10 (0.89–1.36)
3 to < 7	1.60 (1.27–2.00)	1.08 (0.84–1.40)
7 to < 14	1.72 (1.21–2.45)	1.03 (0.71–1.49)
≥ 14	2.48 (1.45–4.22)	1.64 (0.95–2.84)
High happiness		
0	1.00 [Reference]	1.00 [Reference]
< 1	1.29 (0.95–1.76)	1.04 (0.76–1.43)
1 to < 3	1.79 (1.47–2.18)	1.29 (1.04–1.62)
3 to < 7	2.18 (1.73–2.75)	1.57 (1.22–2.03)
7 to < 14	2.51 (1.67–3.78)	1.65 (1.12–2.45)
≥ 14	2.73 (1.38–5.43)	1.83 (0.94–3.56)
Low life stress		
0	1.00 [Reference]	1.00 [Reference]
< 1	1.08 (0.77–1.50)	0.84 (0.60–1.16)
1 to < 3	1.52 (1.22–1.89)	1.14 (0.90–1.43)
3 to < 7	1.71 (1.33–2.19)	1.26 (0.95–1.68)
7 to < 14	1.28 (0.85–1.95)	0.79 (0.51–1.21)
≥ 14	1.53 (0.74–3.19)	1.08 (0.55–2.14)

**Source:** Canadian Health Survey on Children and Youth, 2019. 

**Abbreviations: **CI, confidence interval; h, hour; OPA, outdoor physical activity; OR, odds ratio; wk, week. 

**Notes:** Logistic regression models were used to examine the associations between OPA and the outcome measures. Models were adjusted for age, highest parental education, ethnocultural background, average sleep duration (hours/night), total recreational screen time (hours/week), season, urbanicity and indoor physical activity (categories from no indoor physical activity to ≥ 14 hours/week). 

Positive mental health includes responses of “excellent” and “very good” (vs. “good,” “fair” and “poor”). Low anxiety symptoms and low depressive symptoms include responses of “a few times a year” and “never” (vs. “daily,” “weekly” and “monthly”). High life satisfaction includes scores ≥ 9 on a scale of 0–10 (vs. scores < 9 for low life satisfaction). High happiness includes responses of “happy and interested in life.” Low life stress includes responses of “not at all stressful,” “not very stressful” and “a bit stressful” (vs. “quite a bit stressful” and “extremely stressful”). 

**Table 5:** Association between OPA and mental health, anxiety and depressive symptoms, life satisfaction, happiness and life stress among younger adolescents (12–14 years), Canada, 2019 (n = 5482)

OPA level, h/wk	Unadjusted OR (95% CI)	Adjusted OR (95% CI)
Positive mental health		
0	1.00 [Reference]	1.00 [Reference]
< 1	1.20 (0.87–1.65)	1.22 (0.87–1.72)
1 to < 3	1.31 (1.07–1.60)	1.14 (0.92–1.41)
3 to < 7	1.48 (1.19–1.84)	1.22 (0.97–1.54)
7 to < 14	1.98 (1.43–2.73)	1.53 (1.08–2.16)
≥ 14	2.16 (1.34–3.48)	1.66 (1.00–2.76)
Low anxiety symptoms		
0	1.00 [Reference]	1.00 [Reference]
< 1	0.79 (0.58–1.08)	0.84 (0.61–1.15)
1 to < 3	1.09 (0.89–1.35)	1.12 (0.90–1.40)
3 to < 7	1.06 (0.85–1.33)	1.06 (0.82–1.35)
7 to < 14	0.96 (0.73–1.28)	0.97 (0.72–1.32)
≥ 14	0.85 (0.56–1.29)	0.79 (0.51–1.24)
Low depressive symptoms		
0	1.00 [Reference]	1.00 [Reference]
< 1	0.73 (0.50–1.07)	0.80 (0.53–1.19)
1 to < 3	0.97 (0.74–1.27)	0.95 (0.72–1.26)
3 to < 7	0.92 (0.71–1.21)	0.86 (0.64–1.15)
7 to < 14	0.87 (0.59–1.28)	0.80 (0.53–1.20)
≥ 14	1.14 (0.63–2.06)	1.04 (0.56–1.93)
High life satisfaction		
0	1.00 [Reference]	1.00 [Reference]
< 1	1.32 (0.98–1.78)	1.24 (0.90–1.72)
1 to < 3	1.44 (1.20–1.73)	1.22 (1.00–1.49)
3 to < 7	1.75 (1.42–2.16)	1.44 (1.14–1.82)
7 to < 14	2.10 (1.60–2.76)	1.69 (1.26–2.27)
≥ 14	2.50 (1.68–3.72)	1.74 (1.13–2.68)
High happiness		
0	1.00 [Reference]	1.00 [Reference]
< 1	1.74 (1.29–2.36)	1.70 (1.23–2.36)
1 to < 3	1.54 (1.27–1.88)	1.32 (1.07–1.64)
3 to < 7	2.09 (1.66–2.63)	1.70 (1.32–2.18)
7 to < 14	2.49 (1.84–3.38)	1.90 (1.37–2.62)
≥ 14	4.16 (2.54–6.82)	2.81 (1.71–4.60)
Low life stress		
0	1.00 [Reference]	1.00 [Reference]
< 1	1.02 (0.70–1.49)	1.01 (0.67–1.52)
1 to < 3	1.32 (1.02–1.71)	1.12 (0.85–1.47)
3 to < 7	1.79 (1.29–2.46)	1.41 (0.99–2.00)
7 to < 14	1.53 (1.00–2.34)	1.09 (0.70–1.71)
≥ 14	2.91 (1.38–6.14)	2.08 (0.89–4.82)

Source: Canadian Health Survey on Children and Youth, 2019. 

**Abbreviations: **CI, confidence interval; h, hour; OPA, outdoor physical activity; OR, odds ratio; wk, week. 

**Notes: **Logistic regression models were used to examine the associations between OPA and the outcome measures. Models were adjusted for age, sex, highest parental education, ethnocultural background, average sleep duration (hours/night), total recreational screen time (hours/week), season, urbanicity and indoor physical activity (categories from no indoor physical activity to ≥ 14 hours/week).

Positive mental health includes responses of “excellent” and “very good” (vs. “good,” “fair” and “poor”). Low anxiety symptoms and low depressive symptoms include responses of “a few times a year” and “never” (vs. “daily,” “weekly” and “monthly”). High life satisfaction includes scores ≥ 9 on a scale of 0–10 (vs. scores < 9 for low life satisfaction). High happiness includes responses of “happy and interested in life.” Low life stress includes responses of “not at all stressful,” “not very stressful” and “a bit stressful” (vs. “quite a bit stressful” and “extremely stressful”). 

**Table 6:** Association between levels of OPA and mental health, anxiety and depressive symptoms, life satisfaction, happiness and life stress among older adolescents (15–17 years), Canada, 2019 (n = 4931)

OPA level, h/wk	Unadjusted OR (95% CI)	Adjusted OR (95% CI)
Positive mental health		
0	1.00 [Reference]	1.00 [Reference]
< 1	1.03 (0.73–1.45)	0.87 (0.60–1.27)
1 to < 3	1.33 (1.08–1.64)	1.09 (0.87–1.37)
3 to < 7	1.57 (1.27–1.94)	1.21 (0.95–1.54)
7 to < 14	1.30 (0.91–1.86)	0.94 (0.64–1.38)
≥ 14	2.07 (1.20–3.59)	1.79 (1.00–3.18)
Low anxiety symptoms		
0	1.00 [Reference]	1.00 [Reference]
< 1	1.14 (0.82–1.60)	1.15 (0.80–1.66)
1 to < 3	1.08 (0.87–1.33)	1.06 (0.85–1.33)
3 to < 7	1.35 (1.08–1.68)	1.23 (0.95–1.58)
7 to < 14	1.03 (0.72–1.47)	0.83 (0.56–1.23)
≥ 14	1.18 (0.71–1.95)	1.04 (0.60–1.80)
Low depressive symptoms		
0	1.00 [Reference]	1.00 [Reference]
< 1	1.02 (0.66–1.58)	0.99 (0.65–1.52)
1 to < 3	1.26 (0.98–1.62)	1.17 (0.90–1.52)
3 to < 7	1.38 (1.05–1.82)	1.23 (0.91–1.66)
7 to < 14	1.72 (1.07–2.77)	1.45 (0.84–2.51)
≥ 14	1.01 (0.57–1.80)	0.90 (0.47–1.71)
High life satisfaction		
0	1.00 [Reference]	1.00 [Reference]
< 1	0.94 (0.65–1.35)	0.79 (0.54–1.15)
1 to < 3	1.33 (1.07–1.64)	1.05 (0.83–1.33)
3 to < 7	1.39 (1.13–1.72)	1.06 (0.84–1.35)
7 to < 14	1.60 (1.13–2.28)	1.23 (0.85–1.78)
≥ 14	2.55 (1.50–4.33)	1.87 (1.05–3.31)
High happiness		
0	1.00 [Reference]	1.00 [Reference]
< 1	0.99 (0.71–1.38)	0.87 (0.61–1.22)
1 to < 3	1.83 (1.48–2.25)	1.47 (1.17–1.84)
3 to < 7	2.28 (1.83–2.85)	1.82 (1.42–2.33)
7 to < 14	2.31 (1.60–3.34)	1.83 (1.25–2.67)
≥ 14	2.92 (1.62–5.26)	2.14 (1.13–4.05)
Low life stress		
0	1.00 [Reference]	1.00 [Reference]
< 1	0.96 (0.66–1.40)	0.85 (0.59–1.24)
1 to < 3	1.50 (1.18–1.90)	1.25 (0.98–1.61)
3 to < 7	1.61 (1.27–2.05)	1.25 (0.96–1.63)
7 to < 14	1.19 (0.80–1.77)	0.85 (0.57–1.28)
≥ 14	0.98 (0.55–1.76)	0.70 (0.39–1.24)

**Source: **Canadian Health Survey on Children and Youth, 2019.

**Abbreviations: **CI, confidence interval; h, hour; OPA, outdoor physical activity; OR, odds ratio; wk, week.

**Notes: **Logistic regression models were used to examine the associations between OPA and the outcome measures. Models were adjusted for age, sex, highest parental education, ethnocultural background, average sleep duration (hours/night), total recreational screen time (hours/week), season, urbanicity and indoor physical activity (categories from no indoor physical activity to ≥ 14 hours/week). 

Positive mental health includes responses of “excellent” and “very good” (vs. “good,” “fair” and “poor”). Low anxiety symptoms and low depressive symptoms include responses of “a few times a year” and “never” (vs. “daily,” “weekly” and “monthly”). High life satisfaction includes scores ≥ 9 on a scale of 0–10 (vs. scores < 9 for low life satisfaction). High happiness includes responses of “happy and interested in life.” Low life stress includes responses of “not at all stressful,” “not very stressful” and “a bit stressful” (vs. “quite a bit stressful” and “extremely stressful” for high life stress). 

## Discussion

Using a nationally representative sample of adolescents aged 12 to 17 years living in Canada, we found that OPA was strongly associated with high happiness and high life satisfaction in a dose–response manner. The level of OPA most strongly associated with high happiness and high life satisfaction was 14 or more hours per week (or ≥2 hours/day), which represented the highest exposure category in our analysis. More importantly, the associations were independent of indoor physical activity, suggesting that OPA may provide added benefits to happiness and life satisfaction that indoor physical activity does not provide.

The null associations for anxiety and depressive symptoms and life stress in our study are typical of research in the field,^11^ likely due to the many factors that may contribute to and potentially confound these associations (e.g. quality of OPA, type of outdoor space, interactions with nature, safety of outdoors and so on). However, recent efforts to control for such variables are beginning to show more definitive associations between OPA and health outcomes.^6^,^18^ The cross-sectional nature of previous studies, similar to this study, precludes inferences about causality and temporality.^12^ Several studies have found that cortisol levels decrease when participants spend time in nature, a phenomenon associated with reduced perceived stress.^35^ However, these studies were performed in adult populations, and specifically explored the impact of natural environments on stress.

Higher levels of life satisfaction among adolescents are associated with adaptive psychological functioning, interpersonal and social relationships, academic success, decreased behavioural problems, healthier behaviours (movement, eating and social) and various school-related variables, such as perceived academic efficacy, competence and self-efficacy.^36^ All of these can lead to better mental health outcomes and successful functioning. A scoping review of the health benefits of nature-based physical activity revealed that engaging in OPA, specifically in more natural environments, may have synergistic benefits to mental and physical health compared to physical activity in built environments and indoors.^17^ An important finding of our study was the clear dose–response associations between OPA and high life satisfaction and high happiness. The associations were independent of indoor physical activity, suggesting that OPA may provide added or enhanced benefits. That OPA may provide additional benefits for happiness and life satisfaction compared to indoor physical activity is important for public health guidelines.

Understanding the underlying mechanisms linking OPA to adolescents’ life satisfaction, mental health and happiness can help promote and support OPA. The features of outdoor environments result in specific stimuli that cannot be replicated indoors.^37^ Outdoor environments, with the exposure to sunlight and fresh air, promote a sense of freedom and allow for energetic and exuberant behaviour.^37^ Exposure to sunlight facilitates the secretion of serotonin,^38^ a hormone involved in mood and feelings of happiness and well-being. Outdoor spaces also play a role in encouraging physical activity and promoting social contact between children and youth.^39^ Children and youth prompt each other to be more physically active when they are outdoors, and aspects of outdoor environments (e.g. open spaces, play structures, trees, loose parts) encourage running, walking, climbing, jumping and cycling.^39^,^40^ It is unclear whether the benefits of OPA on mental health can be attributed to physical activity, socialization or some effect of outdoor environments,^41^ but it is likely a combination of these and other factors. Flourishing mental health, being outdoors and physical activity are likely interconnected; spending more time outdoors has been associated with higher levels of physical activity levels, which in turn can increase the probability of flourishing mental health.^15^

Other benefits of increased OPA for children and youth include reduced screen time and improved sleep, both of which can lead to better mental health.^6^,^42^ Although physical activity and socialization can occur indoors, outdoor environments provide a sense of connectedness with nature. In a previous Canadian study, a majority of youth reported that having a connection with nature is important to them, and these youth had reduced psychosomatic symptoms (an indicator of poor mental health).^41^ Averaging more than 0.5 hours per week in nature was associated with a 24% reduction in psychosomatic symptoms among females (with no significant findings for males).^41^ Modelling showed symptom prevalence continuing to decrease until up to 14hours of outdoor play per week.^41^ A systematic review also found numerous impacts of nature connectedness on children’s and youth’s psychological well-being including reduced stress, feelings of joy and happiness, experiences of mindfulness or spirituality and a sense of competence, self-esteem or emotional well-being,^43^ all of which can help improve mental health and life satisfaction.

As previously mentioned, no current guidelines recommend a minimum time for adolescents’ OPA. Our results show that those who spent 14 or more hours per week being active outdoors had the highest prevalence of positive mental health, life satisfaction and happiness. Although 14 hours is by no means a magic number, aiming for this many or more hours of OPA each week (equivalent to 2 hours each day) may be a sensible target given all the potential benefits and the low risk involved. This aligns with the threshold used in the ParticipACTION Report Card.^44^ For some people, having a quantifiable goal provides an amount to strive for and makes the recommendation to replace indoor time with outdoor time less subjective.^11^

***Recommended future research directions***

Future research should aim to clarify the mechanisms by which OPA contributes to higher life satisfaction and happiness among adolescents. Understanding these pathways could inform targeted interventions and mental health strategies. In addition, incorporating objective measures of OPA—such as wearable activity trackers—will improve the accuracy of findings and help validate self-reported data. Longitudinal and intervention studies are needed to establish the directionality of associations and determine whether increasing OPA leads to improved mental health outcomes. Further, research comparing OPA in different settings—urban versus rural and natural versus built environments—could provide valuable insights into how context influences adolescents’ well-being. These findings may ultimately support the development of evidence-based guidelines for adolescent OPA to promote optimal mental and emotional health.

***Strengths and limitations***

Strengths of this study include the large and nationally representative sample, the inclusion of psychometrically valid questions for the dependent variables and noting the significance of these findings for informing future OPA-related strategies. Also, we controlled for indoor physical activity in addition to other covariates, strengthening internal validity and allowing the examination of the added value of OPA on mental health indicators.

The limitations include the subjective nature of the collected variables, the lack of contextual factors (e.g. the quality of OPA, the types of outdoor spaces or their relative safety, interactions with nature, whether the time outdoors is spent alone or with others, and others) and the cross-sectional design, which limits inferences about causality and directionality. Further, residual confounding by unmeasured factors (e.g. pre-existing mental health conditions, chronic illnesses, medication use, social support) is always a possibility in epidemiology. The relatively low response rate (52.1%) could lead to selection bias, where the estimated association between OPA and mental health in the study sample would differ from the estimate had the entire target population agreed to participate. In addition, misclassification of categorical variables is possible, potentially leading to biased estimates of associations or attenuated relationships between exposure and outcome. However, we used the original OPA categories and relied on previously established classifications for the outcome measures.

## Conclusion

OPA was associated with positive mental health, high life satisfaction and high happiness among Canadian adolescents, with levels of OPA of 14 or more hours per week showing the strongest associations. The associations were independent of indoor physical activity and other covariates, suggesting added benefits of OPA on those mental health indicators. Intervention studies that aim to increase OPA are needed to better determine cause-and-effect relationships with various outcomes in the pediatric population.

## Conflicts of interest

Justin Lang is one of this journal’s Associate Scientific Editors and Mark Tremblay is an Editorial Board Member. Both have recused themselves from the review process for this article.

The authors have declared no conflicts of interest.

## Authors’ contributions and statement

JPC: Conceptualization, validation, writing – original draft.

JJL: Formal analysis, validation, writing – review & editing.

SAP: Validation, writing – review & editing.

GSG: Validation, writing – review & editing.

LL: Validation, writing – review & editing.

MST: Validation, writing – review & editing.

TB: Validation, writing – original draft.

All authors gave final approval for the final version and agreed to be accountable for all aspects of the work.

The content and views expressed in this article are those of the authors and do not necessarily reflect those of the Government of Canada.

## Acknowledgements

Jean-Philippe Chaput is supported by the Canadian Institutes of Health Research and the CHEO Research Institute.
